# Assessing Surveillance of Wildlife Diseases by Determining Mammal Species Vulnerability to Climate Change

**DOI:** 10.1155/2023/7628262

**Published:** 2023-08-17

**Authors:** S. R. Wijburg, M. Maas, H. Sprong, A. Gröne, G. van der Schrier, J. M. Rijks

**Affiliations:** ^1^Centre for Infectious Disease Control, National Institute for Public Health and the Environment (RIVM), 3720 BA Bilthoven, Netherlands; ^2^Dutch Wildlife Health Centre, Division of Pathology, Department of Biomolecular Health Sciences, Faculty of Veterinary Medicine, University of Utrecht, Yalelaan 1, 3584 CL, Utrecht, Netherlands; ^3^Royal Netherlands Meteorological Institute (KNMI), 3731 GA De Bilt, Netherlands

## Abstract

Climate change is one of the drivers of wildlife-borne disease emergence, as it can affect species abundance and fitness, host immunocompetence, and interactions with pathogens. To detect emerging wildlife-borne diseases, countries may implement general wildlife-disease surveillance systems. Such surveillance exists in the Netherlands. However, it is unclear how well it covers host species vulnerable to climate change and consequently disease emergence in these species. Therefore, we performed a trait-based vulnerability assessment (TVA) to quantify species vulnerability to climate change for 59 Dutch terrestrial mammals. Species' vulnerability was estimated based on the magnitude of climatic change within the species' distribution (exposure), the species' potential to persist *in situ* (sensitivity), and the species' ability to adjust (adaptive capacity). Using these vulnerability categories, we identified priority species at risk for disease emergence due to climate change. Subsequently, we assessed the frequency of occurrence of these priority species compared to other mammal species examined in general wildlife disease surveillance during 2008–2022. We identified 25% of the mammal species to be highly exposed, 24% to be highly sensitive, and 22% to have a low adaptive capacity. The whiskered myotis and the garden dormouse were highly vulnerable (i.e., highly exposed, highly sensitive, and low adaptive capacity), but they are rare in the Netherlands. The Western barbastelle, the pond bat, and the Daubenton's myotis were potential adapters (highly exposed, highly sensitive, and high adaptive capacity). Species vulnerable to climate change were relatively poorly represented in current general surveillance. Our research shows a comprehensive approach that considers both exposures to climate change and ecological factors to assess vulnerability. TVAs, as presented in this study, can easily be adapted to include extra drivers and species, and we would therefore recommend surveillance institutes to consider integrating these types of assessments for evaluating and improving surveillance for wildlife-borne disease emergence.

## 1. Introduction

Climate change, largely caused by anthropogenic changes on the planet, is one of the major health threats for both humans and animals [1, 2]. Under climate change, various species must adapt to their changed environment or relocate to new environments for subsistence [3–5]. Throughout this process, host immunocompetence can be affected [6]. Additionally, changes in species assemblages may occur, resulting in new interactions between hosts and microorganisms [5, 7–9]. Thus, shifts in the abundance of microorganisms and invasions of novel microorganisms into previously unexposed or more resilient host populations may result in changes in disease dynamics [9] and may potentially facilitate the emergence of wildlife-borne zoonotic diseases.

Fluctuations in the occurrence of wildlife diseases, including zoonoses, can be detected through disease surveillance. Surveillance systems can focus on detecting a specific disease or pathogen (targeted surveillance) or any disease or pathogen (general surveillance) [10]. General wildlife disease surveillance often relies on investigating unusual wildlife mortality incidents through postmortem examination of carcasses. This monitoring is based on the voluntary reporting and collection of dead wild animal specimens, and given their intrinsic nature, only a limited number of wildlife mortality incidents can be investigated through this kind of surveillance system. It is currently a black box how the wildlife sample surveyed in general disease surveillance relates to specific threats and drivers of wildlife-borne emerging infectious (zoonotic) diseases, such as climate change. Therefore, additional metrics for assessing the general surveillance data relative to such drivers of disease are needed.

Because the degree of exposure to climate change, the ability of a species to persist in their current environment, and the potential of species to accommodate themselves to the shifting climate may partly determine the rise of (emerging) wildlife diseases, trait-based vulnerability assessments (TVA) can be used to identify host species of concern. A TVA is a framework that gives insight into what extent species appear to be affected by environmental changes like climate change (threats) [11]. TVAs are based on the hypothesis that a species' capability to deal with the impact of a threat depends on both external and internal factors, such as the level of exposure to the perturbation and their life history traits that allow a species to cope with or adapt to this exposure [11, 12]. This capability or absence thereof can be quantified as “species vulnerability” to the threat [13]. The dimensions of vulnerability typically include exposure (i.e., degree of change to a species' environment by the threat), species sensitivity (i.e., species potential to persist *in situ*), and species adaptive capacity (i.e., species' ability to deal with the impacts of a driver or to relocate) [11, 14]. Up to now, several studies have investigated the vulnerability of species to climate change on global or continental scales [11, 15–19]. However, few have conducted them on a national level for surveillance purposes [20–22].

Here, we determined the host species' vulnerability to climate change to understand the level of coping required from the mammals represented in our surveillance sample and discuss possible implications for the surveillance of emerging diseases. To achieve this, we used a TVA to identify the climate change vulnerability of wild mammal species at the scale of the Netherlands and then evaluated the relative representation of the vulnerability categories in the Dutch general wildlife disease surveillance program from 2008 to 2022 (by assessing the frequency of occurrence of these priority species relative to other mammal species examined from 2008 to 2022). First, we quantified exposure by determining the degree of climate change within the geographical range of Dutch mammals [11, 15]. Then we considered a combination of traits (e.g., ecological, behavioral, and biological) that could affect the level of sensitivity of a species or bestow species with lesser or greater adaptive capacity regarding the impact of climate change. These three components combined were used to classify Dutch mammalian wildlife in terms of vulnerability to climate change [21]. Finally, we analyzed the sample of mammals examined through the general wildlife disease surveillance system in the Netherlands in 2008–2022 in terms of species vulnerability to climate change and discussed possible implications.

## 2. Materials and Methods

### 2.1. Vulnerability Assessment

**Figure 1:**
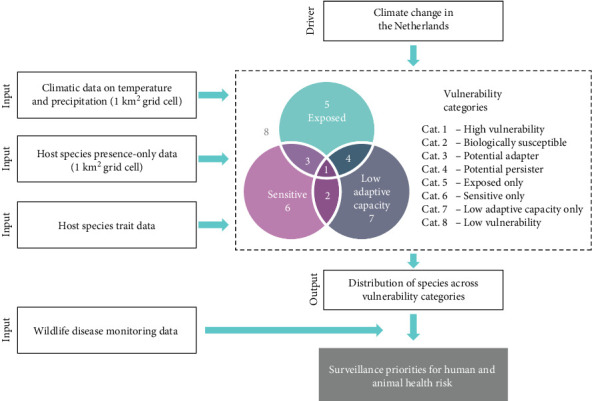
Adapted framework for the assessment of the impact of national climate change on mammals according to the IUCN methodology [11], additionally illustrating the link toward surveillance priorities for human and animal health risk. Mammals scoring high across all dimensions (exposure, sensitivity, and low adaptive capacity) are classified as highly vulnerable (1). Biologically susceptible mammals (2) are not exposed but have a high sensitivity and a low adaptive capacity. Potential adapters (3) are exposed and sensitive but have a high adaptive capacity, and potential persisters (4) are exposed and have a low adaptive capacity but have a low sensitivity to climate change [11]. Species not occurring in any of these four categories were classified as “exposed only” (5), “sensitive only” (6), “low adaptive capacity only” (7), or “low vulnerability” (low risk in all dimensions of vulnerability; 8) [11].

As a first step, a list was compiled of all terrestrial mammals that are considered native to the Netherlands (circa 50.6°N to 53.7°N Latitude, 3.2°E to 7.4°E Longitude) [23] (Table S1). We then followed the TVA framework, explained in detail by Foden et al. [11] (Figure 1) to assess the three dimensions (i.e., exposure to climate change, sensitivity, and adaptive capacity) of vulnerability to climate change for these mammal species. In this approach, mammals that are, for instance, highly sensitive and bestowed with a low adaptive capacity were referred to as “biologically susceptible (Category 2)” to the shifting climate [16]. Biologically susceptible mammals, which additionally have endured high exposure to climate change, were considered to have a high vulnerability to climate change (Category 1). Mammals which were highly sensitive and highly exposed, but have a high adaptive capacity were potential adapters (Category 3) [11, 16]. We assumed climate likely has the greatest direct effect on health in species belonging to Categories 1 and 3, due to the potential impact on immunocompetence, species abundance, and host pathogen contact.

### 2.2. Assessment Exposure TVA Dimension

To determine the magnitude of climate change within the Netherlands, we used a 30-year base period (1961–1990) as a reference, a timeframe duration recommended by the World Meteorological Organization [24]. Following the methodology described by Williams et al. [25], climate dissimilarities between the baseline period (average from 1961 to 1990) and the recent period (average from 1991 to 2020) were quantified by calculating the standardized euclidean distances (SED) per grid cell (1 km^2^) as follows:

(1) $$\begin{array}{c} \text{SED/grid cell}={\sqrt{\sum }}_{k=1}^{n=6}\frac{{\left(b_{ki}\;-a_{kj}\right)}^{2}}{s_{kj}^{2}}. \end{array}$$

In this formula, *n* is the number of included climatic variables (here *n* = 6; Table 1), *a* is the mean of climate parameter *k* for the baseline period (1961–1990) at grid cell *j*, *b* is the mean climate for the recent period (1991–2020) at grid cell *i*, and *s*_*kj*_ is the standard deviation (SD) of the interannual variability for the baseline period [25]. Six climate metrics related to temperature and precipitation were selected (Table 1): annual mean temperature (BIO01, °C), maximum temperature of the warmest month (BIO05, °C), minimum temperature of the coldest month (BIO06, °C), annual precipitation (BIO12, mm), precipitation of the wettest month (BIO13, mm), and precipitation of the driest month (BIO14, mm) [26–28]. These parameters were selected as they display a general trend of temperature and precipitation means and extremes [27], and were not found to be collinear (Pearson's *r* < 0.7 [29], Table S2). In above mentioned formula, all climatic variables are standardized to place them on a common scale and to emphasize on trends that are relatively large compared to historic interannual variability [25, 30].

To determine the degree of exposure to climate change at the species level, we first retrieved presence-only data (i.e., a sample with only observed presence and unknown absences), from 2008 until 2020, on the selected terrestrial mammalian wildlife species (data provided by the Dutch Mammal Society). These data were used to generate a database containing the presence per grid cell with a spatial resolution of 1 km × 1 km (36,799 grid cells, excluding sea grid cells). Differences in detectability between species were not considered. We then divided the range of SED values into sections by use of the tercile of the entire range of SED values. The upper category was classified as 2, the middle category as 1, and the lowest category as 0 [15]. For each species' geographical distribution, the number of cells occurring per category were counted and used to determine the exposure value [15]:

(2) $$\begin{array}{c} \text{Exposure}=a\times 0+b\times 1+c\times 2. \end{array}$$

In this formula, *a*, *b*, and *c* are the percentage of cells in each category (e.g., lower, middle, upper) [15]. Resulting exposure values were standardized by max–min linear rescaling [28] as follows:

(3) $$\begin{array}{c} \frac{x_{i}-x_{\text{⁣}\min}}{x_{\text{⁣}\max}-x_{\text{⁣}\min}}. \end{array}$$

Species in the upper 25% were classified as exposed to climate change [11]. An example of the determination of the exposure value has been provided in Method S1 and Figure S4.

### 2.3. Assessment Sensitivity and Adaptive Capacity TVA Dimension

To characterize mammals' sensitivity and adaptive capacity to climate change, we selected four traits pertaining to high sensitivity (i.e., (1) body mass; (2) fossoriality; (3) diurnality; (4) habitat specialism) [14, 19, 31–33] and identified four traits as important factors affecting a mammal's adaptive capacity (i.e., (1) dispersal distance; (2) diet specialism; (3) reproductive capacity; (4) generation length) [3, 14, 18, 19, 31–35]. Data on dispersal distance were missing for all species of the order Chiroptera. For these species, spatial behavior (i.e., sedentary migrants (<10 km), regional migrants (10–100 km), or long-distance migrants (>100 km)) was therefore taken as a proxy [36, 37]. Traits and their hypothesized impact in relation to climate change vulnerability are outlined in Table 2.

To offer a quantitative ranking of species' sensitivity and adaptive capacity to climate change, we followed the methodology described by Albouy et al. [15]. All traits were given equal weights. Each trait was evaluated using a three-point scale, with two being the most sensitive and zero being the least sensitive, two having the lowest adaptive capacity, and zero having the highest [15]. Traits described by categorical values were scored according to categorical thresholds [15, 19]. For instance, species inhabiting five or more [50] classification type one habitats were scored as zero, species inhabiting between two and four [50] habitats were scored as one, and species inhabiting only one habitat type were scored as two (Table S4). In the case of traits expressed as continuous variables, categorization was done by using the tercile of the range [15]. To assess the robustness of the thresholds selected for continuous traits, an additional statistical sensitivity test was performed [15]. This test was done by moving either the first break or the second break of the initial tercile categorization toward the minimum or maximum values. The amount by which the breaks were moved varied between 1% and 33%. To assess the impact of moving the breaks, the Pearson correlation between the initial classification and a given scenario was determined (Method S2, Figure S5) [15]. Overall sensitivity and adaptive capacity scores were obtained by summation of all trait values (each between 0 and 2). The resulting values were standardized by max–min linear rescaling [28]. Species were classified as highly sensitive when they were among the 25% species with the highest overall sensitivity scores. Species were categorized as having a low adaptive capacity when they belonged to the 25% species with the highest adaptive capacity ranking.

### 2.4. General Surveillance System in the Netherlands

To determine the number of mammal species investigated in general wildlife disease surveillance, we used a dataset from the Dutch Wildlife Health Centre (DWHC) containing records between January 2008 and August 2022. All records of completely or partially necropsied specimens were included in this study, provided there was valid location data (i.e., longitude and latitude), and they were not collected for a specific (targeted) research project. Complete necropsy referred to macroscopical and histological examination of at least five of the six following key organs of the specimen: brain, heart, lungs, liver, spleen, and kidneys, and sometimes with cytological examination of the following organs: lung, liver, and spleen. Specimens were classified as partially necropsied when two or more key organs were missing or were too autolytic for histological assessment, when the specimen was only assessed on a macroscopical level, or when essential body parts of the specimen were absent (e.g., head). See Table S3 for the description of all examination levels used at the DWHC.

### 2.5. Software Used

Spatial data extraction and analyses were conducted in R version 4.1.2. Spyder (Python 3.8) from the anaconda navigator software was used for the retrieval of climate data (KNMI, https://dataplatform.knmi.nl/).

## 3. Results

Our TVA initially included 60 terrestrial mammalian species (Table S1). The European water vole (*Arvicola sherman*) was excluded preceding the assessment because occurrence data were not available. The final sample included species of the orders Chiroptera (30.5%, 18/59), Rodentia (27.1%, 16/59), Carnivora (16.9%, 10/59), Eulipotyphla (13.5%, 8/59), Cetartiodactyla (8.5%, 5/59), and Lagomorpha (3.4%, 2/59).

### 3.1. Assessment Exposure TVA Dimension

**Figure 2:**
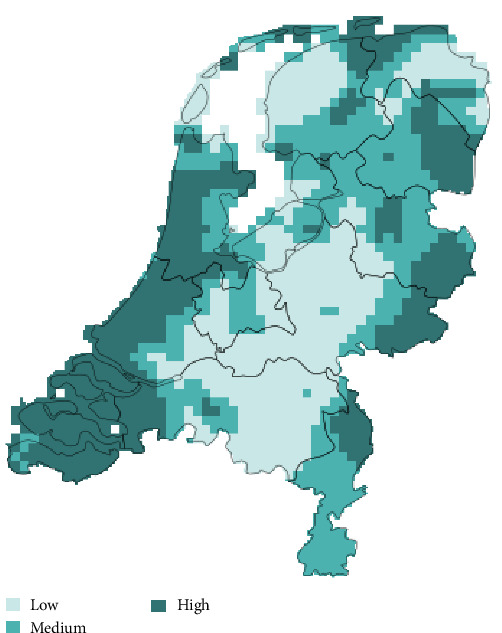
Extent of climate change in the Netherlands per 25 km^2^. Areas in the Netherlands that are experiencing the highest degree of change between the baseline period (1961–1990) and the recent period (1991–2020) are shown in dark turquoise (SED ≥ 2.33), areas with a medium degree of change are displayed in turquoise (2.20 ≤ SED < 2.33), and regions with a low amount of change shown in light turquoise (SED < 2.20). Degree of change per included bioclimatic factor is shown in Figure S2.

**Figure 3:**
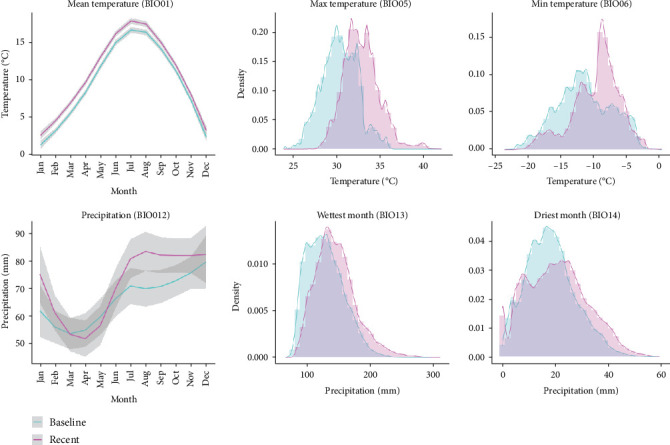
Bioclimatic predictors of baseline and recent periods. BIO01, average monthly temperature in the baseline period versus in the recent period (confidence interval (CI) is displayed in gray). BIO05, the maximum temperature of the warmest month in the baseline period versus the recent period. BIO06, minimum temperature of the coldest month in the baseline period versus the recent period. BIO12, monthly precipitation in the baseline period versus the recent period (CI is displayed in gray). BIO13, precipitation in the wettest month in the baseline period versus the recent period. BIO14, precipitation in the driest month in the baseline period versus the recent period. Precipitation is measured in millimeters (mm), and temperature is expressed in degrees Celsius (°C).

The mean magnitude of climate change, quantified as SEDs, was 2.28 and ranged from 1.99 to 2.84 (SD = 0.14). Spatially, the overall magnitude of climate change appeared to be most prominent in the coastal areas (Figure 2). Furthermore, based on the graphs used to quantify climate change within the Netherlands, a climatic shift between the baseline (1961–1990) and the recent period (1991–2020) was recognizable with a trend towards higher temperatures (BIO01, BIO05, BIO06) (Figure 3). Additionally, both the yearly amount of precipitation (BIO12) and the amount of precipitation in the wettest month (BIO13) increased throughout the recent period. Finally, a decrease in the total amount of precipitation in the driest month was detected when comparing the recent period to the baseline period.

The climate change exposure value, standardized to a range between 0.00 and 1.00, had a median value of 0.45 (IQR: 0.08) for the species assessed. Considering the exposure cutoff value of 0.5 (i.e., low exposure > 0.5 ≥ high exposure), 15 species were highly exposed (25.4%, 15/59). More specifically, 50% (4/8) of the Eulipotyphla, 33% (6/18) of the Chiroptera, 25% (4/16) of the Rodentia, and 10% (1/10) of the Carnivora were classified as highly exposed to climate change within their geographical ranges (based on presence-only data). A Chiroptera, namely, the Western barbastelle (*Barbastella barbastellus*), was the most exposed species (Exposure = 1). It was followed by an Eulipotyphla, the bicolored shrew (*Crocidura leucodon*) (Exposure = 0.91).

### 3.2. Assessment Sensitivity and Adaptive Capacity TVA Dimension

Nearly 24% (14/59) of the assessed species were classified as highly sensitive to climate change. These consisted of 61% (11/18) of the Chiroptera and 19% (3/16) of the Rodentia. A low body mass and not being adapted to digging and life underground (i.e., fossoriality) were the traits that contributed relatively most to the species being deemed highly sensitive (Figure S3).

Furthermore, 22% of the species were scored to have a low adaptive capacity. Of these, the order of Chiroptera additionally contained the highest percentage of species with a low adaptive capacity (44%, 8/18), followed by the order Eulipotyphla (37.5%, 3/8), Cetartiodactyla (20%, 1/5), and Rodentia (6%, 1/16) (Table 3).

### 3.3. Assessment Vulnerability

**Figure 4:**
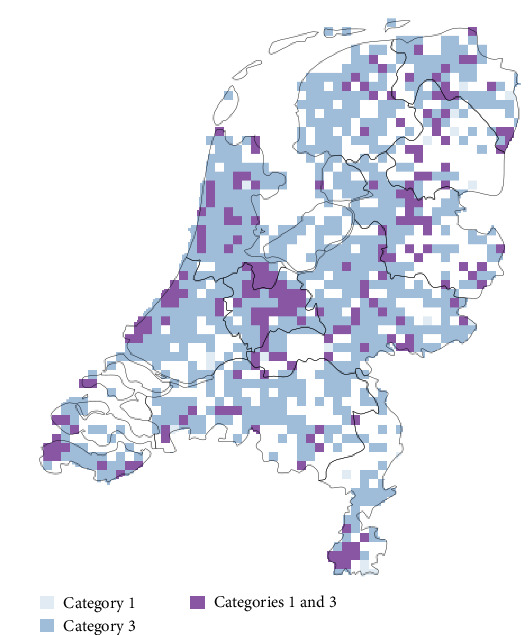
The presence of species classed as highly vulnerable (Category 1) or as potential adapters (Category 3) per 25-square-kilometer block.

A full breakdown of the vulnerability assessment, in accordance with the eight climate change vulnerability categories, is presented in Table 3. Under this framework, two species were classed as highly vulnerable to climate change: the whiskered myotis (*Myotis mystacinus*) and the garden dormouse (*Eliomys quercinus*) (Category 1). Five species were categorized as biologically susceptible (Category 2), three species as potential adapters (Category 3), and two species as potential persisters (Category 4). More than half of the species (31/59) fell into Category 8 (i.e., low vulnerability). The spatial occurrence of Category 1 or 3 species, in which climate change is likely to have the greatest direct effect on health, is shown in Figure 4.

### 3.4. Overview of the DWHC General Wildlife Disease Surveillance System

**Figure 5:**
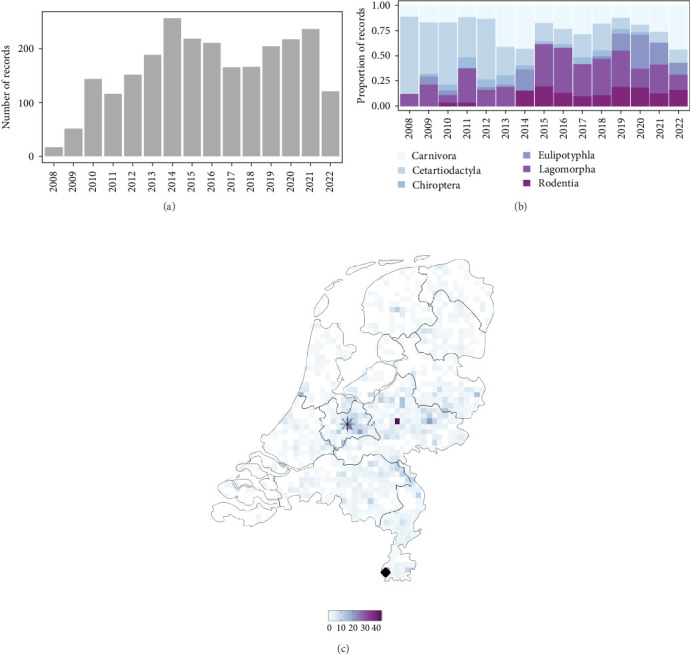
Number of carcasses of wild mammals per year (sampling year 2022 is incomplete) received by DHWC. The peak submission year was in 2014 (*n* = 256) (a). The relative records examined per mammalian order (b) and the geographical location from which the records originated (c). The location of the DWHC is indicated by the black star (c). The two locations of the category 1 species in the DWHC database were found 3.2 kilometers from each other and are together indicated by a diamond.

Between 2008 and 2022, the DWHC received a total of 3560 dead wild mammals (36 host species) to be investigated through postmortem examination (Table S3). Within this timeframe, we identified 69.2% (2,463/3,560) records that were classified as completely or partially necropsied and contained valid spatial coordinate data (mean 164 ± 67.2 records/year (min = 17, max = 256); Figure 5(a)). Mammals from the order Lagomorpha (671/2,463, 27.2%) were most often sent to the DWHC, followed by mammalian species from the orders Carnivora (615/2,463, 25.0%), the Cetartiodactyla (594/2,463, 24.1%), the Rodentia (271/2,463, 11%), the Eulipotyphla (205/2,463, 8.3%), and from species of the order Chiroptera (107/2,463, 4.3%) (Figure 5(b)). Geographically, most investigated dead wild mammals originated from the middle of the Netherlands (Figure 5(c)) and not necessarily from the coastal areas in which climate dissimilarities were greatest (Figure 2).

**Figure 6:**
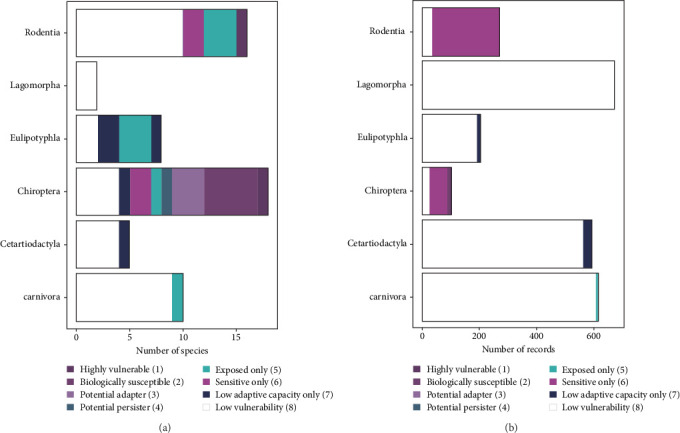
The number of species per order per vulnerability category (a) and the cumulative number of records per order within the DWHC database (b, Table S1).

The species categorized as highly vulnerable (Category 1) or as a potential adapter (Category 3) belonged to the orders Chiroptera and Rodentia (Figure 6(a)). The proportion of Category 8 species belonged mostly to the orders Lagomorpha, Cetartiodactyla, and Carnivora (Figure 6(a)). Most dead wild mammals received by the DWHC were also mostly Category 8 species (Figure 6(b)). This indicates that Category 1 and 3 species are relatively poorly represented in the DWHC database sample. More specifically, the garden dormouse and the whiskered myotis, both Category 1 species, were not or only present twice, respectively. Additionally, no Category 3 species were completely or partially necropsied between 2008 and 2022.

## 4. Discussion

This study implemented a TVA for climate change at a national level and linked it to general wildlife surveillance to identify animal species of surveillance priority. We detected a heterogenous pattern both in species' presence distribution, as well as in the spatial degree of climate change. Around 25% of the mammalian species were found to have experienced a relatively high degree of exposure to climate change in the last 30 years, nearly 24% of the species possessed traits that made them highly sensitive, and 22% were bestowed with a low adaptive capacity.

A driver, such as climate change, may alter the diversity and composition of local animal communities due to such differences in vulnerability scores (e.g., range shifts, altered relative abundance by changes in death and/or birth rates) [12, 52–54]. Climate change might act as a long-term stressor causing physiological responses in species that are exposed to climate change, cannot accommodate to it, and have difficulty to persist *in situ* when exposed (highly vulnerable species, Category 1; the garden dormouse, and the whiskered myotis) [55, 56]. Species that are exposed to climate change and have difficulty to persist *in situ* may have higher initial adaptability (potential adapters, Category 3; Western barbastelle, the Pond bat, and the Daubenton's myotis), but this might imply they are forced to change their geographical distribution. This shift in species distribution is still difficult to predict [57, 58] and can impose additional challenges, such as contact with novel pathogens and may result in shifting disease dynamics and (negative) health outcomes for the host (e.g., changing species assemblages, host-pathogen interactions, and altered interactions with endemic pathogens and hosts) [9, 59–61]. This has repercussions for pathogen transmission [53, 62, 63], for example, illustrated by exacerbated declines (e.g., wild meerkats (*Suricata suricatta*) in Kalahari [64]) and species extinction (e.g., the Monteverde harlequin frog (*Atelop*us sp.) and the golden toad (*Bufo periglenes*) in Costa Rica [65]) as a result of climate change driven diseases. The disappearance of vulnerable host species from a community could, in addition, offer opportunities to those less vulnerable.

Two out of the 59 Dutch mammal species were highly vulnerable to climate change (Category 1): the garden dormouse and the whiskered myotis. Both species are rare in the Netherlands [66]. The Dutch Mammal Society has listed the garden dormouse as critically endangered. This species has an estimate of 50 reproducing individuals in three atlas blocks (grid cells of 25 km^2^) and a distribution that has declined with 77% since the 1950s [66]. The whiskered myotis, with an estimated 1000 adult animals reproducing in 134 atlas blocks, was classified as vulnerable; its numbers have declined with 38% in the last 10 years in the Netherlands [66]. Cited causes underlying these declines include the disturbance and disappearance of suitable habitats and the continued decline of flying insect populations [66]. Stress in relation to climate change can be expected in both highly vulnerable species, making them also more prone to infection and disease [67]. Nevertheless, even if climate change enhanced disease emergence in one of these species in the Netherlands, the small population sizes make it questionable if this could progress within the Dutch populations into an emerging infectious disease of more general concern. The small numbers also make it unlikely that either species will be found dead and submitted for surveillance in the Netherlands. However, if submitted, a thorough investigation of such cases is warranted, with possibly the use of metagenomics for the detection of untargeted pathogens [68].

Three out of the 59 Dutch mammal species were classified as potential adapters to climate change (Category 3): the Western barbastelle (*Barbastella barbastellus barbastellus*), the pond bat (*Myotis dasycneme*), and the Daubenton's myotis (*Myotis daubentonii*). The Western barbastelle was classified as regionally extinct because there is currently no indication that this species procreates within the Netherlands [66]. The Pond bat is considered endangered in the Netherlands because its population of an estimated 4,500 adult animals is fragmented, reproduces only in 65 atlas blocks, and has decreased by 32% in the last decade [66]. Daubenton's myotis is classified as a species of least concern (an estimated number of 15,000 adult animals reproducing in 454 atlas blocks). Bat species are generally sensitive to environmental changes [69]. Both the pond bat and Daubenton's myotis species might show an adaptive response to climate change as changes in temperature and rainfall patterns are expected to continue (e.g., wetter winters, higher temperatures, more intense rain showers, and higher chances of drier summers) [70]. Their high adaptive response is not related to reproductive rate and generation length [69]. Rather, it is related to high dispersal capacity, making it easier for these species to move to novel environments. While both bat species are still rare, they are more common than the highly vulnerable whiskered myotis, and they occur in higher densities. We conclude that both species of bats are interesting for further detection of infectious (zoonotic) diseases emergence because of climate change in the Netherlands. While their numbers make it more likely that they are found dead and are submitted for general disease surveillance than the whiskered myotis, a longitudinal surveillance program may also be considered for detecting changes in pathogen composition in these Category 3 species.

Evaluation of the current wildlife disease surveillance by the DWHC from 2008 to 2022 showed a discrepancy between the species frequently submitted to the DWHC (i.e., lagomorphs, carnivores, and ungulates; Figure 6(b)) and the species allocated most into the vulnerability categories (i.e., bats, rodents; Figure 6(a)). This discrepancy suggests that species that are more susceptible to climate change are less well represented in the general surveillance database. However, the two highly vulnerable species (i.e., Category 1) occur in such low numbers that detection is limited from the beginning. General wildlife surveillance offers an ideal setting for emerging wildlife disease discovery. Yet the underrepresentation of certain species and geographical sample locations is, and will continue to be a problem [71, 72]. For a carcass to reach a surveillance institute, it needs to persist in the environment, it needs to be detected and reported in time, and it needs to be delivered to the institute [72]. The persistence of the carcass in the environment differs per species and is often surprisingly brief [73]. Moreover, potential differences arising because of reporting bias (i.e., the chance that a detected species is reported to the DWHC) should also be considered [72]. The reporting of a carcass is dependent on initial detection by citizens. Consequently, this process is controlled by what the public perceives as a valuable species (e.g., game versus non-game species) and their perceived need to submit the carcass (e.g., single case versus mass mortality, pathogen spread awareness) [71, 72].

This paper has demonstrated that one can assess wildlife vulnerability with accurate data on geographical distribution and on ecological traits. This improved understanding of potential vulnerability in relation to a driver may enable wildlife health surveillance institutes to focus surveillance efforts relating to emerging wildlife disease discovery. However, inherent to any TVA, our study comes with a degree of uncertainty: (i) a driver might not affect every mammalian species in the same manner, thereby introducing uncertainties in the underlying assumptions about the traits [16, 19, 74–77]; (ii) species ranges might have changed, and population measurement programs between various species differ. This may have led to over- or underestimating the actual degree of exposure to the driver within the chosen time window; (iii) while conducting the assessment, the possibility that either species' sensitivity and/or their adaptive capacity may vary over time was not addressed [19]; (iv) the degree of species' vulnerability to the impacts of a driver is strongly attributable to the included traits, selected species, and underlying available data [21, 78]. The traits included here were based on the previous studies, but they were only a few, making each one weigh heavily. This means that the outcomes of TVAs represent relative vulnerability scores, which makes it hard to meaningfully compare them with other studies [11, 79]. In addition, as species vulnerability is not universally defined, different classification systems are available depending on exposure, sensitivity, or adaptive capacity. Many of these use different thresholds to classify species into vulnerability categories [11, 12, 14, 15, 41]. Future research should therefore clarify which method and which ecological characteristics will lead to the best predictions of climate change vulnerability.

To reduce these causes of uncertainty, empirical validation of the framework and the ecological robustness of the assessment in future work are essential (e.g., case–control field studies) [80]. Future developments can further broaden the scope of this study by incorporating pathogens' traits associated with (changes in) infection risk and/or disease emergence. Describing the effect of a driver across all participating species in pathogen transmission is crucial in understanding zoonotic risks. Similarly, our analysis included only the driver of climate change, although species are rarely threatened by one driver [81]. The cumulative risk presented by the presence of multiple drivers could, however, be relatively easily constructed based on the methods provided within this paper. Finally, our analysis focused on mammal species, but bird species and their interactions might additionally be an important target for risk predictions, especially with the ongoing Avian influenza epidemic and the impact a driver can have on successful migration [82–85].

## 5. Conclusions

Improving wildlife health surveillance is challenging for several reasons. Ecosystems are changing due to the synergetic impact of many drivers across temporal, organizational, and spatial scales [86]. In addition, the fundamental biology of host, vector, and pathogens continues to be an understudied field, especially in the context of novel anthropogenic changes presented to species. By using a TVA, we were able to enhance and contextualize our understanding on how a driver is likely to affect a species in a certain area of interest. We determined that two species are potentially highly vulnerable to climate change relative to other Dutch wild mammals (Category 1). Additionally, we were able to identify three sensitive and exposed species (Category 3). Because these species are likely coping with the changing climate situation by adapting to potential disturbances in ecological balances, this may result in (new) wildlife diseases. Consequently, we think that using a TVA to determine the impact of a driver can serve as a starting point of guiding current surveillance strategies and may help refining hypotheses, even though validation via field studies remains essential. The TVA presented in this study is a tool that could be adapted to include extra drivers (e.g., pollution and urbanization) and species (e.g., birds and pathogens), and we would therefore recommend surveillance institutes to consider integrating these kinds of assessments.

## Figures and Tables

**Table 1:** Bioclimatic predictors^a^ [26].

Metrics	Unit	Description
BIO01, annual mean temperature	Degrees Celsius (°C)	The annual mean temperature was calculated based on the monthly mean temperature
BIO05, maximum temperature of the warmest month	Degrees Celsius (°C)	The maximum temperature recorded in the warmest month for a given year
BIO06, minimum temperature of the coldest month	Degrees Celsius (°C)	The minimum temperature recorded in the coldest month for a given year
BIO12, annual precipitation	Millimeters (mm)	Annual precipitation is calculated by summation of total monthly precipitation values
BIO13, precipitation of the wettest month	Millimeters (mm)	The total precipitation recorded within the wettest month
BIO14, precipitation of the driest month	Millimeters (mm)	The total precipitation recorded within the driest month

*Note*: ^a^Interpolated data from 34 weather stations in the Netherlands were obtained on a 1 km^2^ spatial resolution as raster layers from the Royal Netherlands Meteorological Institute (KNMI, https://dataplatform.knmi.nl/). These raster layers were used to extract values for daily precipitation and daily mean, minimum, and maximum temperatures.

**Table 2:** Traits and their underlying hypothesized effects on species sensitivity and adaptive capacity in relation to climate change^a^.

Category	Traits	Description	Hypothesized impact of climate change
Sensitivity	Body mass	Adult mass (in grams)	Bigger species are more robust physically and so may be less sensitive to climate change. A bigger body mass is generally associated with higher energy reserves, which thus decreases sensitivity to shortage of food [19, 38]
	Fossorial	Yes or no, yes referred to mammal species being adapted to digging and life underground	Species that display fossorial behavior may be less likely to be at risk of climate change due to their ability to find shelter from extreme temperatures [33, 39]
	Diurnality	Yes or no, yes referred to mammal species being diurnal only	Mammals that are only active throughout the daytime are believed to be more exposed to extreme temperatures and more likely to be at risk of climate change [19, 40]
	Habitat specialism	Number of distinct levels of one habitat type occupied by a mammal^b^	A specialized mammal is more tightly dependent on specific environmental requirements and conditions and is, therefore, more likely to be at risk of climate change [16, 31, 33, 41]

Adaptive capacity	Dispersal distance	The distance traveled between the place of birth and the place of reproduction (in kilometers)	Species with a low dispersal ability are more likely to be at risk of climate change, as these species might not be able to move to novel suitable environments [3, 31, 34, 41]
	Diet specialism	Number of level 1 and level 2 dietary items eaten by a species^c^	Species with broader dietary breadths are assumed to have a higher ability to utilize resources and to establish themselves in novel areas [31–33]
	Reproductive capacity	Number of offspring maximally produced by a mammal [42]. Calculated according to: RC = ((*L*_max_ − *L*_1*r*_)^*∗*^LS^*∗*^LPY) In which: (i) RC: reproductive capacity (ii) *L*_max_: maximum lifespan (iii) *L*_1r_: age first reproduction (iv) LS: litter size (v) LPY: number of litters per year	Species with a high reproductive output will be less affected by the shifting climate as they will produce a sufficient number of offspring to compensate for potential population losses [17, 18, 32, 43–45]
	Generation length	The average age of parents of the current cohort (in days)	Reflects the turnover rate of breeding individuals within a population [46]. Longer generation lengths have been demonstrated to be associated with a heightened risk of extinction under climate change [32, 35]

*Note*: Categorization of species traits using a three-point scale is displayed in Table S4. ^a^For our analysis, trait data were gathered using published literature [36] and online databases (IUCN Red List (IUCN), COMBINE [47], MammalDiet2 [48, 49]). ^b^[50] classes of level one habitat types: forest, savanna, shrubland, grassland, wetlands (inland), rocky areas (e.g., inland cliffs, mountain peaks), caves & Subterranean Habitats (nonaquatic), desert, marine neritic, marine oceanic, marine intertidal, marine coastal/supratidal, artificial—terrestrial, artificial—aquatic, introduced vegetation, and other (Figure S1) ([51]). ^c^Dietary items: invertebrates, mammals, birds, herptiles, fish, woody (browser), herbaceous (grazer), seeds, fruit, nectar, roots, and other (buds/flowers/pollen/gum/fungi/lichens) (Figure S1). The items most abundant in the diet of mammals were classified as level 1, dietary items regularly consumed by a mammal but in a lower amount were classified as level 2 dietary items, dietary items rarely consumed are classified as level 3, and level 0 was ascribed to dietary items not recorded in the diet of a species [48, 49].

**Table 3:** Summary of vulnerability scores in mammals relative to climate change according to the TVA framework (Figure 1) [11].

Vulnerability type	High exposure	High sensitivity	Low adaptive capacity	*N* (%)	Species name^a^	Order
1. Highly vulnerable				2 (3.4)	Whiskered myotis Garden dormouse	Chiroptera Rodentia

2. Biologically susceptible				5 (8.5)	Bechstein's myotis Brown long-eared bat Geoffroy's bat Gray long-eared bat Soprano pipistrelle	Chiroptera Chiroptera Chiroptera Chiroptera Chiroptera

3. Potential adapter				3 (5.1)	Daubenton's myotis Pond bat Western barbastelle	Chiroptera Chiroptera Chiroptera

4. Potential persister				2 (3.4)	Eurasian pygmy shrew Greater mouse-eared bat	Eulipotyphla Chiroptera

5. Exposed only				8 (13.6)	Stoat Brandt's myotis Bicolored shrew Crowned shrew Greater white-toothed shrew Yellow-necked field mouse European pine vole Tundra vole	Carnivora Chiroptera Eulipotyphla Eulipotyphla Eulipotyphla Rodentia Rodentia Rodentia

6. Sensitive only				4 (6.8)	Common pipistrelle Nathusius' pipistrelle Eurasian red squirrel Hazel dormouse	Chiroptera Chiroptera Rodentia Rodentia

7. Low adaptive capacity only				4 (6.8)	Fallow deer Natterer's bat Eurasian water shrew European mole	Cetartiodactyla Chiroptera Eulipotyphla Eulipotyphla

8. Low vulnerability				31 (52.5)	Beech marten Eurasian badger Eurasian otter Gray wolf Least weasel Pine marten Red fox Western polecat Wildcat European bison Red deer Roe deer Wild boar Common noctule Lesser noctule Particolored bat Serotine bat Common shrew European hedgehog European hare Rabbit Bank vole Black rat Brown rat Common hamster Common vole Eurasian beaver Eurasian harvest mouse Field vole House mouse Long-tailed field mouse	Carnivora Carnivora Carnivora Carnivora Carnivora Carnivora Carnivora Carnivora Carnivora Cetartiodactyla Cetartiodactyla Cetartiodactyla Cetartiodactyla Chiroptera Chiroptera Chiroptera Chiroptera Eulipotyphla Eulipotyphla Lagomorpha Lagomorpha Rodentia Rodentia Rodentia Rodentia Rodentia Rodentia Rodentia Rodentia Rodentia Rodentia

Total number of mammal species				**59**		

*Note*: ^a^Scientific names of included species are presented in Table S1.

## Data Availability

Historical and current climate data are available at https://dataplatform.knmi.nl/. Data on species diet breadth is available at https://doi.org/10.1111/mam.12119. Data on the dispersal of species of the Chiroptera order was retrieved from the report “Action Plan for the Conservation of All Bat Species in the European Union 2018–2024” [7]. Data about species' adult body mass, fossoriality, diurnality, habitat breadth, dispersal distance, maximum lifespan, age first reproduction, litter size, and the number of litters per year is available at https://doi.org/10.1002/ecy.3344. Data on the geographical distribution of species are available at https://www.verspreidingsatlas.nl/. Surveillance data provided by the DWHC are available in Table S1.

## Supplementary Materials

Supplementary Materials Figure S1: Dietary categories: invertebrates, mammals, birds, herptiles, fish, woody (browser), herbaceous (grazer), seeds, fruit, nectar, roots, and other (buds/flowers/pollen/gum/fungi/lichens). The items most abundant in the diet of mammals were classified as level 1, dietary items regularly consumed by a mammal but in a lower amount were classified as level 2 dietary items, dietary items rarely consumed are classified as level 3, and level 0 was ascribed to dietary items not recorded in the diet of a species [28, 41]. [36] classification type 1 habitats: forest, savanna, shrubland, grassland, wetlands (inland), rocky areas (e.g., inland cliffs, mountain peaks), caves & Subterranean Habitats (non-aquatic), desert, marine neritic, marine oceanic, marine intertidal, marine coastal/supratidal, artificial – terrestrial, artificial – aquatic, introduced vegetation, and other ([37]). Figure S2: Areas in the Netherlands that are experiencing the highest degree of change between the baseline period (1961 – 1990) and the recent period (1991 – 2020) are shown in dark turquoise, areas with a medium degree of change are displayed in turquoise, and regions with a low amount of change in those shown in light turquoise. Figure S3: Proportion of species per trait per category for sensitivity (0 = low sensitivity, 1 = medium sensitivity, 2 = high sensitivity; Figure S3A). Proportion of species per trait per category for adaptive capacity (0 = high adaptive capacity, 1 = medium adaptive capacity, 2 = low adaptive capacity; Figure S3B). Figure S4: Calculated degree of climate change within the geographical range of the Whiskered Myotis (Figure S4A) and the Geoffrey's Bat (Figure S4B). Colours indicate the degree of climate dissimilarity (low, medium, and upper) between the baseline (1961 – 1990) and the recent period (1991 – 2020). The black points on the map visualize the geographical range of the Whiskered Myotis and the Geoffrey's Bat. The barcharts show of the number of cells in which the species is present per category of climate dissimilarity. Figure S5: Pearson correlation between the sensitivity and adaptive capacity results of the initial scenario and the calculated sensitivity and adaptive scores based on given scenarios. On the y-axis, the amount by which the breaks were moved is shown between 1% and 33%. On the x-as, the multiple scenarios are shown. Namely, moving of the first break towards the maximum (break 1-max), moving of the first break towards the minimum (break 1-min), moving of the second break towards the maximum (break 2-max), and moving of the second break towards the minimum (break 2-max) [2]. Table S1: Ranking of included mammals according to their vulnerability category (i.e., potential adapter (PA), potential persister (PP), high latent risk (HLR), high vulnerability (HV), sensitive only (SO), low adaptive capacity only (LACO), and low vulnerability (LV)) and their abundance in the DWHC database. Table S2: Pearson correlation between the bioclimatic variables during the baseline (above the diagonal line) and the recent period (below the diagonal line). Table S3: Definitions of necropsy levels and the number of records per necropsy level within the DWHC database. Table S4: Categorisation of species traits using a three-point scale. Each trait was evaluated based on their mechanisms to heighten sensitivity and to lower the adaptive capacity of species. Trait values were summed per species, which resulted in an overall sensitivity and adaptive capacity ranking. Method S1: Calculated degree of climate dissimilarity (low, medium, and upper) between the baseline (1961–1990) and the recent period (1991–2020) within geographical range of two species. Method S2: Sensitivity analysis of the sensitivity and adaptive capacity index.
