# Supplementary material for: Assessing Surveillance of Wildlife Diseases by Determining Mammal Species Vulnerability to Climate Change
Source: Transbound Emerg Dis. 2023 Aug 17;2023:7628262. doi: 10.1155/2023/7628262 (PMC12016766; doi:10.1155/2023/7628262)
Supplement: Supplementary Materials — Figure S1: Dietary categories: invertebrates, mammals, birds, herptiles, fish, woody (browser), herbaceous (grazer), seeds, fruit, nectar, roots, and other (buds/flowers/pollen/gum/fungi/lichens). The items most abundant in the diet of mammals were classified as level 1, dietary items regularly consumed by a mammal but in a lower amount were classified as level 2 dietary items, dietary items rarely consumed are classified as level 3, and level 0 was ascribed to dietary items not recorded in the diet of a species [28, 41]. [36] classification type 1 habitats: forest, savanna, shrubland, grassland, wetlands (inland), rocky areas (e.g., inland cliffs, mountain peaks), caves & Subterranean Habitats (non-aquatic), desert, marine neritic, marine oceanic, marine intertidal, marine coastal/supratidal, artificial – terrestrial, artificial – aquatic, introduced vegetation, and other ([37]). Figure S2: Areas in the Netherlands that are experiencing the highest degree of change between the baseline period (1961 – 1990) and the recent period (1991 – 2020) are shown in dark turquoise, areas with a medium degree of change are displayed in turquoise, and regions with a low amount of change in those shown in light turquoise. Figure S3: Proportion of species per trait per category for sensitivity (0 = low sensitivity, 1 = medium sensitivity, 2 = high sensitivity; Figure S3A). Proportion of species per trait per category for adaptive capacity (0 = high adaptive capacity, 1 = medium adaptive capacity, 2 = low adaptive capacity; Figure S3B). Figure S4: Calculated degree of climate change within the geographical range of the Whiskered Myotis (Figure S4A) and the Geoffrey's Bat (Figure S4B). Colours indicate the degree of climate dissimilarity (low, medium, and upper) between the baseline (1961 – 1990) and the recent period (1991 – 2020). The black points on the map visualize the geographical range of the Whiskered Myotis and the Geoffrey's Bat. The barcharts show of the number of cells i [file 7628262.f1.docx]

# Supporting information

# Supplemental figures


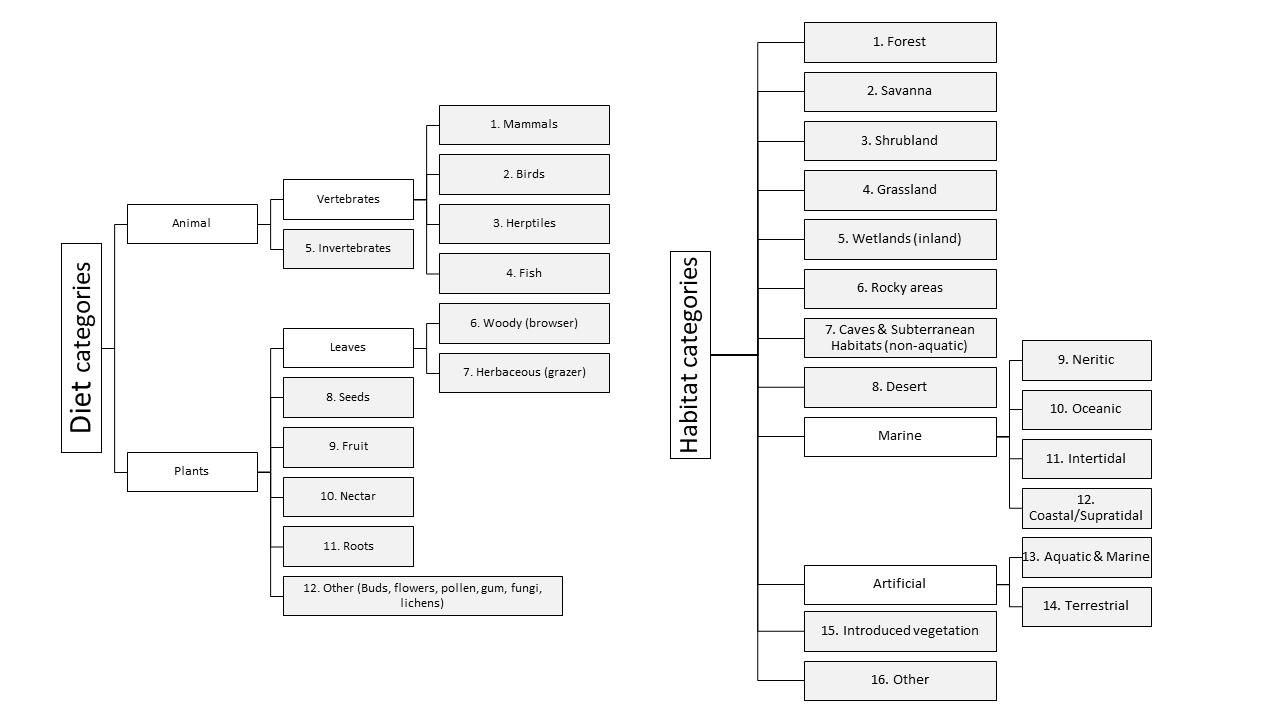


**Figure S1.** Dietary categories: invertebrates, mammals, birds, herptiles, fish, woody (browser), herbaceous (grazer), seeds, fruit, nectar, roots, and other (buds/flowers/pollen/gum/fungi/lichens). The items most abundant in the diet of mammals were classified as level 1, dietary items regularly consumed by a mammal but in a lower amount were classified as level 2 dietary items, dietary items rarely consumed are classified as level 3, and level 0 was ascribed to dietary items not recorded in the diet of a species (Gainsbury, Tallowin, & Meiri, 2018; Kissling et al., 2014). IUCN classification type 1 habitats: forest, savanna, shrubland, grassland, wetlands (inland), rocky areas (e.g., inland cliffs, mountain peaks), caves & Subterranean Habitats (non-aquatic), desert, marine neritic, marine oceanic, marine intertidal, marine coastal/supratidal, artificial – terrestrial, artificial – aquatic, introduced vegetation, and other (IUCN).

*
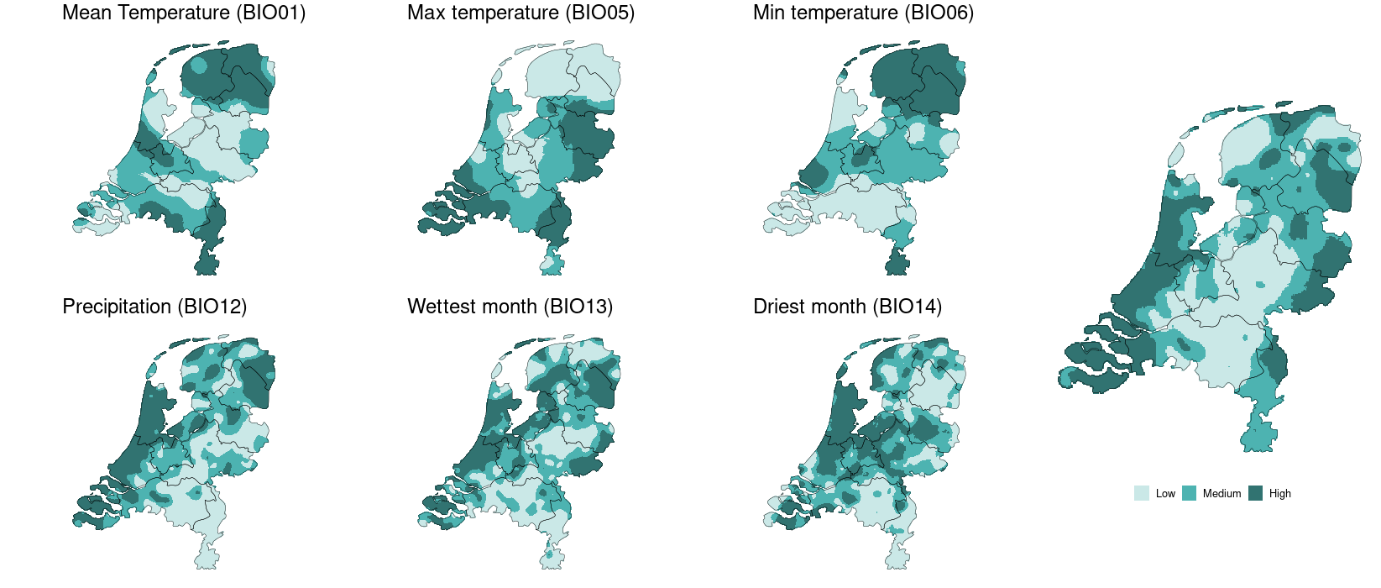
*

**Figure S****2. Extent of climate change in the Netherlands**. Areas in the Netherlands that are experiencing the highest degree of change between the baseline period (1961 – 1990) and the recent period (1991 – 2020) are shown in dark turquoise, areas with a medium degree of change are displayed in turquoise, and regions with a low amount of change in those shown in light turquoise.


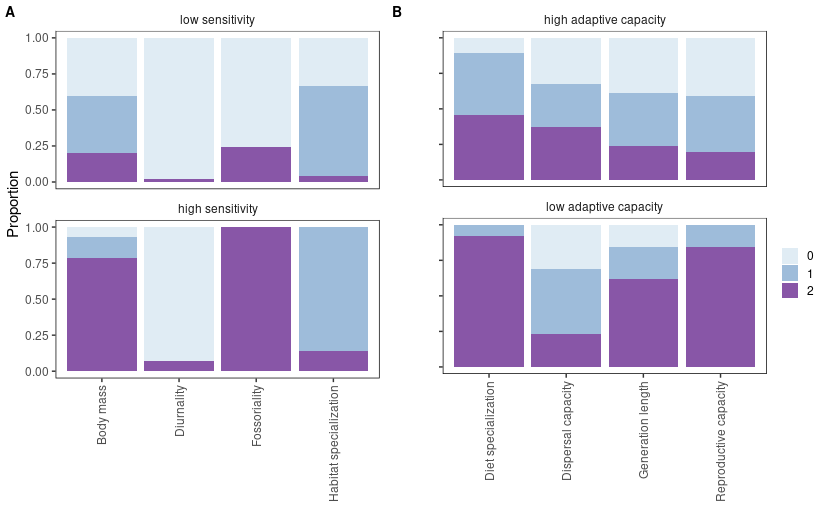


**Figure S3.** Figure S3A. Proportion of species per trait per category for sensitivity (0 = low sensitivity, 1 = medium sensitivity, 2 = high sensitivity). Figure S3B. Proportion of species per trait per category for adaptive capacity (0 = high adaptive capacity, 1 = medium adaptive capacity, 2 = low adaptive capacity).

**Supplemental tables**

**Table S1**. Ranking of included mammals according to their vulnerability category (i.e., potential adapter (PA), potential persister (PP), high latent risk (HLR), high vulnerability (HV), sensitive only (SO), low adaptive capacity only (LACO), and low vulnerability (LV)) and their abundance in the DWHC database.

| Order | Family | Scientific name | English name | Exposure | Sensitivity | Adaptive capacity | Vulnerability category | # DWHC database | Source – Native to the Netherlands |
| --- | --- | --- | --- | --- | --- | --- | --- | --- | --- |
| Carnivora | *Canidae* | *Canis lupus* | Grey wolf | 0.25 | 0 | 0.4 | LV | 12 | (IUCN) |
| Carnivora | *Canidae* | *Vulpes vulpes* | Red fox | 0.43 | 0 | 0 | LV | 131 | (IUCN) |
| Carnivora | *Felidae* | *Felis silvestris* | Wildcat | 0.45 | 0.2 | 0.2 | LV | 1 | (IUCN) |
| Carnivora | *Mustelidae* | *Lutra lutra* | Eurasian otter | 0.42 | 0 | 0.6 | LV | 13 | (IUCN) |
| Carnivora | *Mustelidae* | *Martes foina* | Beech marten | 0.38 | 0.2 | 0.2 | LV | 42 | (IUCN) |
| Carnivora | *Mustelidae* | *Martes martes* | Pine marten | 0.37 | 0.2 | 0.2 | LV | 8 | (IUCN) |
| Carnivora | *Mustelidae* | *Meles meles* | Eurasian badger | 0.32 | 0.6 | 0.2 | LV | 324 | (IUCN) |
| Carnivora | *Mustelidae* | *Mustela erminea* | Stoat | 0.54 | 0.2 | 0.2 | EO | 8 | (IUCN) |
| Carnivora | *Mustelidae* | *Mustela nivalis* | Least weasel | 0.46 | 0.2 | 0.4 | LV | 45 | (IUCN) |
| Carnivora | *Mustelidae* | *Mustela putorius* | Western polecat | 0.45 | 0 | 0 | LV | 31 | (IUCN) |
| Cetartiodactyla | *Bovidae* | *Bison bonasus* | European bison | 0.44 | 0.2 | 0.6 | LV | 6 | (IUCN) |
| Cetartiodactyla | *Cervidae* | *Capreolus capreolus* | Roe deer | 0.42 | 0.2 | 0.6 | LV | 493 | (IUCN) |
| Cetartiodactyla | *Cervidae* | *Cervus elaphus* | Red deer | 0 | 0.2 | 0.6 | LV | 30 | (IUCN) |
| Cetartiodactyla | *Cervidae* | *Dama dama* | Fallow deer | 0.48 | 0.2 | 0.8 | LACO | 32 | (NDFF) |
| Cetartiodactyla | *Suidae* | *Sus scrofa* | Wild boar | 0.13 | 0 | 0 | LV | 33 | (IUCN) |
| Chiroptera | *Vespertilionidae* | *Barbastella barbastellus* | Western barbastelle | 1 | 1 | 0.6 | PA | 0 | (IUCN) |
| Chiroptera | *Vespertilionidae* | *Eptesicus serotinus* | Serotine bat | 0.42 | 0.6 | 0.6 | LV | 23 | (IUCN) |
| Chiroptera | *Vespertilionidae* | *Myotis bechsteinii* | Bechstein's myotis | 0.45 | 1 | 0.8 | HLR | 0 | (IUCN) |
| Chiroptera | *Vespertilionidae* | *Myotis brandtii* | Brandt's myotis | 0.69 | 0.8 | 0.6 | EO | 0 | (IUCN) |
| Chiroptera | *Vespertilionidae* | *Myotis dasycneme* | Pond bat | 0.51 | 1 | 0.6 | PA | 0 | (IUCN) |
| Chiroptera | *Vespertilionidae* | *Myotis daubentonii* | Daubenton's Myotis | 0.5 | 1 | 0.6 | PA | 0 | (IUCN) |
| Chiroptera | *Vespertilionidae* | *Myotis emarginatus* | Geoffroy's bat | 0.38 | 1 | 1 | HLR | 1 | (IUCN) |
| Chiroptera | *Vespertilionidae* | *Myotis myotis* | Greater Mouse-eared Bat | 0.53 | 0.8 | 0.8 | PP | 0 | (IUCN) |
| Chiroptera | *Vespertilionidae* | *Myotis mystacinus* | Whiskered myotis | 0.52 | 1 | 0.8 | HV | 2 | (IUCN) |
| Chiroptera | *Vespertilionidae* | *Myotis nattereri* | Natterer’s bat | 0.38 | 0.8 | 1 | LACO | 2 | (IUCN) |
| Chiroptera | *Vespertilionidae* | *Nyctalus leisleri* | Lesser noctule | 0.43 | 0.8 | 0.6 | LV | 0 | (IUCN) |
| Chiroptera | *Vespertilionidae* | *Nyctalus noctula* | Common noctule | 0.43 | 0.8 | 0.6 | LV | 1 | (IUCN) |
| Chiroptera | *Vespertilionidae* | *Pipistrellus nathusii* | Nathusius' pipistrelle | 0.48 | 1 | 0.6 | SO | 4 | (IUCN) |
| Chiroptera | *Vespertilionidae* | *Pipistrellus pipistrellus* | Common pipistrelle | 0.44 | 1 | 0.6 | SO | 58 | (IUCN) |
| Chiroptera | *Vespertilionidae* | *Pipistrellus pygmaeus* | Soprano pipistrelle | 0.46 | 1 | 0.8 | HLR | 0 | (NDFF) |
| Chiroptera | *Vespertilionidae* | *Plecotus auritus* | Brown long-eared bat | 0.37 | 1 | 0.8 | HLR | 14 | (IUCN) |
| Chiroptera | *Vespertilionidae* | *Plecotus austriacus* | Grey Long-eared Bat | 0.33 | 1 | 0.8 | HLR | 2 | (IUCN) |
| Chiroptera | *Vespertilionidae* | *Vespertilio murinus* | Particoloured bat | 0.43 | 0.8 | 0.6 | LV | 0 | (IUCN) |
| Eulipotyphla | *Erinaceidae* | *Erinaceus europaeus* | European hedgehog | 0.45 | 0.2 | 0.4 | LV | 182 | (IUCN) |
| Eulipotyphla | *Soricidae* | *Crocidura leucodon* | Bicoloured Shrew | 0.91 | 0.6 | 0.6 | EO | 0 | (NDFF) |
| Eulipotyphla | *Soricidae* | *Crocidura russula* | Greater white-toothed Shrew | 0.51 | 0.8 | 0.6 | EO | 3 | (IUCN) |
| Eulipotyphla | *Soricidae* | *Neomys fodiens* | Eurasian water shrew | 0.47 | 0.6 | 0.8 | LACO | 0 | (IUCN) |
| Eulipotyphla | *Soricidae* | *Sorex araneus* | Common shrew | 0.50 | 0.6 | 0.6 | LV | 9 | (IUCN) |
| Eulipotyphla | *Soricidae* | *Sorex coronatus* | Crowned Shrew | 0.52 | 0.6 | 0.6 | EO | 0 | (IUCN) |
| Eulipotyphla | *Soricidae* | *Sorex minutus* | Eurasian Pygmy Shrew | 0.53 | 0.4 | 0.8 | PP | 0 | (NDFF) |
| Eulipotyphla | *Talpidae* | *Talpa europaea* | European mole | 0.46 | 0.4 | 0.8 | LACO | 11 | (IUCN) |
| Lagomorpha | *Leporidae* | *Lepus europaeus* | European hare | 0.46 | 0.2 | 0.2 | LV | 550 | (IUCN) |
| Lagomorpha | *Leporidae* | *Oryctolagus cuniculus* | Rabbit | 0.45 | 0.2 | 0.2 | LV | 121 | (NDFF) |
| Rodentia | *Castoridae* | *Castor fiber* | Eurasian beaver | 0.30 | 0.2 | 0.4 | LV | 26 | (NDFF) |
| Rodentia | *Cricetidae* | *Cricetus cricetus* | Common hamster | 0.45 | 0.2 | 0.2 | LV | 0 | (IUCN) |
| Rodentia | *Cricetidae* | *Microtus agrestis* | Field vole | 0.45 | 0.2 | 0.2 | LV | 0 | (IUCN) |
| Rodentia | *Cricetidae* | *Microtus arvalis* | Common vole | 0.49 | 0.4 | 0.4 | LV | 0 | (IUCN) |
| Rodentia | *Cricetidae* | *Microtus oeconomus* | Tundra vole | 0.66 | 0.4 | 0.6 | EO | 0 | (IUCN) |
| Rodentia | *Cricetidae* | *Microtus subterraneus* | European pine vole | 0.62 | 0.4 | 0.4 | EO | 0 | (IUCN) |
| Rodentia | *Cricetidae* | *Myodes glareolus* | Bank vole | 0.44 | 0.8 | 0.4 | LV | 1 | (IUCN) |
| Rodentia | *Gliridae* | *Eliomys quercinus* | Garden Dormouse | 0.60 | 1 | 0.8 | HV | 0 | (IUCN) |
| Rodentia | *Gliridae* | *Muscardinus avellanarius* | Hazel dormouse | 0.45 | 1 | 0.4 | SO | 0 | (IUCN) |
| Rodentia | *Muridae* | *Apodemus flavicollis* | Yellow-necked field mouse | 0.63 | 0.4 | 0.6 | EO | 0 | (NDFF) |
| Rodentia | *Muridae* | *Apodemus sylvaticus* | Long-tailed field mouse | 0.46 | 0.6 | 0.2 | LV | 4 | (IUCN) |
| Rodentia | *Muridae* | *Micromys minutus* | Eurasian harvest mouse | 0.49 | 0.4 | 0.4 | LV | 0 | (IUCN) |
| Rodentia | *Muridae* | *Mus musculus* | House mouse | 0.48 | 0.6 | 0.2 | LV | 1 | (IUCN) |
| Rodentia | *Muridae* | *Rattus norvegicus* | Brown rat | 0.5 | 0.6 | 0 | LV | 0 | (NDFF) |
| Rodentia | *Muridae* | *Rattus rattus* | Black rat | 0.37 | 0.8 | 0 | LV | 2 | (NDFF) |
| Rodentia | *Sciuridae* | *Sciurus vulgaris* | Eurasian red squirrel | 0.41 | 1 | 0.4 | SO | 237 | (IUCN) |

**Table S2.** Pearson correlation between the bioclimatic variables during the baseline (above the diagonal line) and the recent period (below the diagonal line).

|  | BIO01 | BIO05 | BIO06 | BIO12 | BIO13 | BIO14 |
| --- | --- | --- | --- | --- | --- | --- |
| BIO01 |  | 0.35 | 0.66 | -0.044 | -0.098 | -0.002 |
| BIO05 | 0.24 |  | 0.0063 | -0.33 | -0.29 | 0.034 |
| BIO06 | 0.6 | -0.0064 |  | 0.085 | 0.044 | -0.024 |
| BIO12 | 0.2 | -0.32 | 0.29 |  | 0.68 | 0.34 |
| BIO13 | -0.048 | -0.27 | 0.19 | 0.59 |  | 0.039 |
| BIO14 | 0.048 | -0.026 | 0.035 | 0.36 | -0.055 |  |

**Table S3.** Definitions of necropsy levels and the number of records per necropsy level within the DWHC database.

| Type of surveillance | Necropsy level | Definition | # in DWHC database |
| --- | --- | --- | --- |
| General surveillance | Complete Necropsy | Macroscopic and histologic assessment of the specimen must contain five out of six key organs^#^. Cytology most contain two out of three organs^¥^. | 1891^^^ |
| General surveillance | Complete Necropsy minus cytology | Macroscopic and histologic assessment of the specimen must contain five out of six key organs^#^. Cytology was not (sufficiently) assessed (e.g., artifacts due to freezing, autolyze, and/or contamination). | 418 |
| General surveillance | Partial necropsy | a. Two or more key organs are missing and/or autolytic in the histologic assessment of the specimen. | 232^^^ |
|  |  | b. Specimen has been assessed only on a macroscopical level. Histology and cytology were not performed. |  |
|  |  | c. Specimen is missing essential body parts (e.g., head). |  |
| Other | No necropsy | a. Specimen has been frozen for future examination. | 851 |
|  |  | b. Specimen is too autolytic for further examination upon arrival. |  |
|  |  | c. Specimen has been sent to the WBVR without being examined by the DWHC. |  |
| Other | Other | a. Laboratory tests only: only specific samples of the specimen were examined for laboratory testing (e.g., sampling for tularemia, COVID-19, Usutu, etc.). | 168 |
|  |  | b. Tissue: Specimen of which only one or more organs, blood or tissue have been received by the DWHC. |  |
|  |  | c. Project: Specimen collected for research projects |  |

^#^ Key organs: Brain, heart, lungs, liver, kidneys, and spleen.

^¥^ Cytological slides: HC liver, HC spleen, and HC lung.

^^^ One complete necropsied specimen and two partially necropsied specimen contained invalid spatial coordinate data and were therefore excluded from the final dataset.

**Table S4.** Categorisation of species traits using a three-point scale. Each trait was evaluated based on their mechanisms to heighten sensitivity and to lower the adaptive capacity of species. Trait values were summed per species, which resulted in an overall sensitivity and adaptive capacity ranking.

| Components | Traits | Categories |  |  |
| --- | --- | --- | --- | --- |
|  |  | **0** | **1** | **2** |
| Sensitivity | Body mass (log-transformed) | 2.52 – 5.80 | 1.20 – 2.45 | 0.623 - 1.18 |
|  | Diurnality | No |  | Yes |
|  | Habitat specialization | ≥ 5 | 2 ≥ 4 | 1 |
|  | Fossoriality | Yes |  | No |
| Adaptive capacity | Dispersal capacity (log-transformed) | 4 – 5 | 3.09 – 4 | 1.85 – 2.92 |
|  | Diet specialism | ≥ 5 | 2 ≥ 4 | 1 |
|  | Reproductive output (log-transformed) | 1.76 – 2.53 | 1.83 – 1.74 | 0.95 – 1.38 |
|  | Generation length | 220 – 875 | 1,000 – 2,179 | 5,209 – 2,195 |

**Methods**

**Method S1.** Calculated degree of climate dissimilarity (low, medium, and upper) between the baseline (1961 – 1990) and the recent period (1991 – 2020) within geographical range of two species.

Here we present an example of the determination of the exposure value of two species belonging to the Genus *Myotis*. Both the whiskered myotis (*Myotis mystacinus*), the Geoffroy's bat (*Myotis emarginatus*), were highly sensitive and bestowed with a low adaptive capacity but showed a contrast in exposure to climate change within their geographical ranges. The whiskered myotis had an overall exposure value of 0.52. In comparison to the Geoffroy's bat which had an overall exposure value of 0.38, as in the last decades this species was mainly present in the south (NDFF). This indicated that, in the last 30 years, the whiskered myotis endured a higher degree of climatic dissimilarities within its ranges.


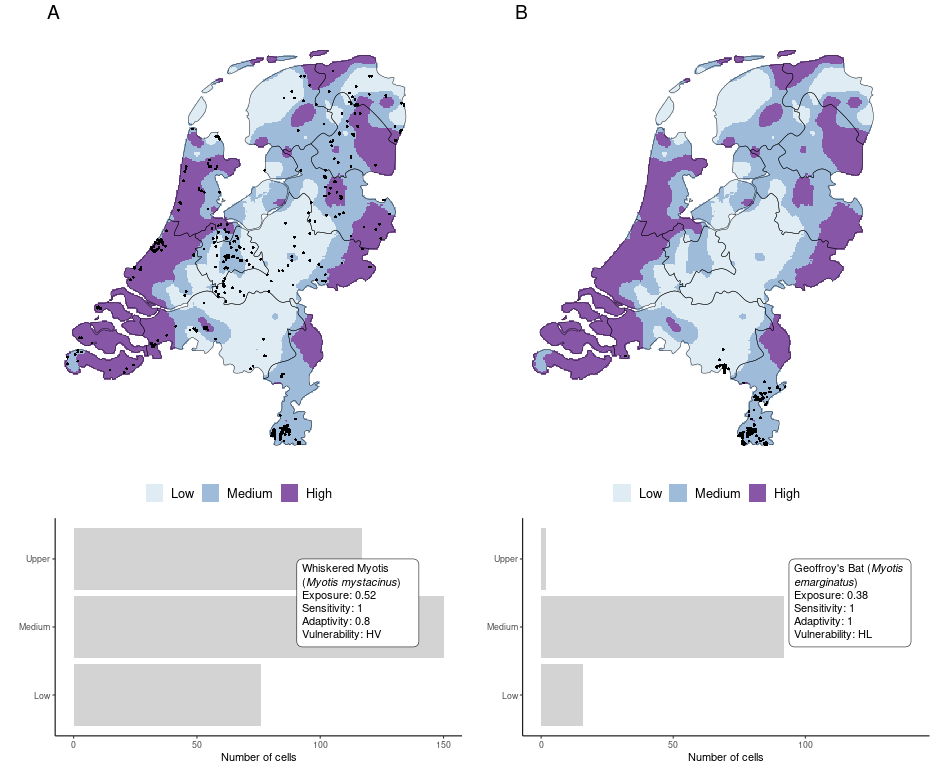


**Figure S4**. Calculated degree of climate change within the geographical range of the Whiskered Myotis (Figure S4A) and the Geoffrey’s Bat (Figure S4B). Colours indicate the degree of climate dissimilarity (low, medium, and upper) between the baseline (1961 – 1990) and the recent period (1991 – 2020). The black points on the map visualize the geographical range of the Whiskered Myotis and the Geoffrey’s Bat. The barcharts show of the number of cells in which the species is present per category of climate dissimilarity.

**Method S2**. Sensitivity analysis of the sensitivity and adaptive capacity index.

For each trait, expressed as a continuous variable, the tercile of the range was calculated. We followed the methodology described by Albouy et al. to evaluate the robustness of these breaks (Albouy et al., 2020). In short, this was done by moving the either the first or the second break towards the minimum (scenario break 1-min, scenario break 2-min) or maximum (scenario break 1-max, scenario break 2-max) value of the range. The amount by which the breaks were moved for each given scenario ranged between 1% and 33%. The Pearson correlation between the initial classification and a scenario is shown in Figure S3. Correlation values ranged between 0.96 and 1.00 for the sensitivity scenarios and between 0.83 and 1.00 for the adaptive capacity scenarios. These outcomes indicate that both the sensitivity and adaptive capacity indexes are robust.


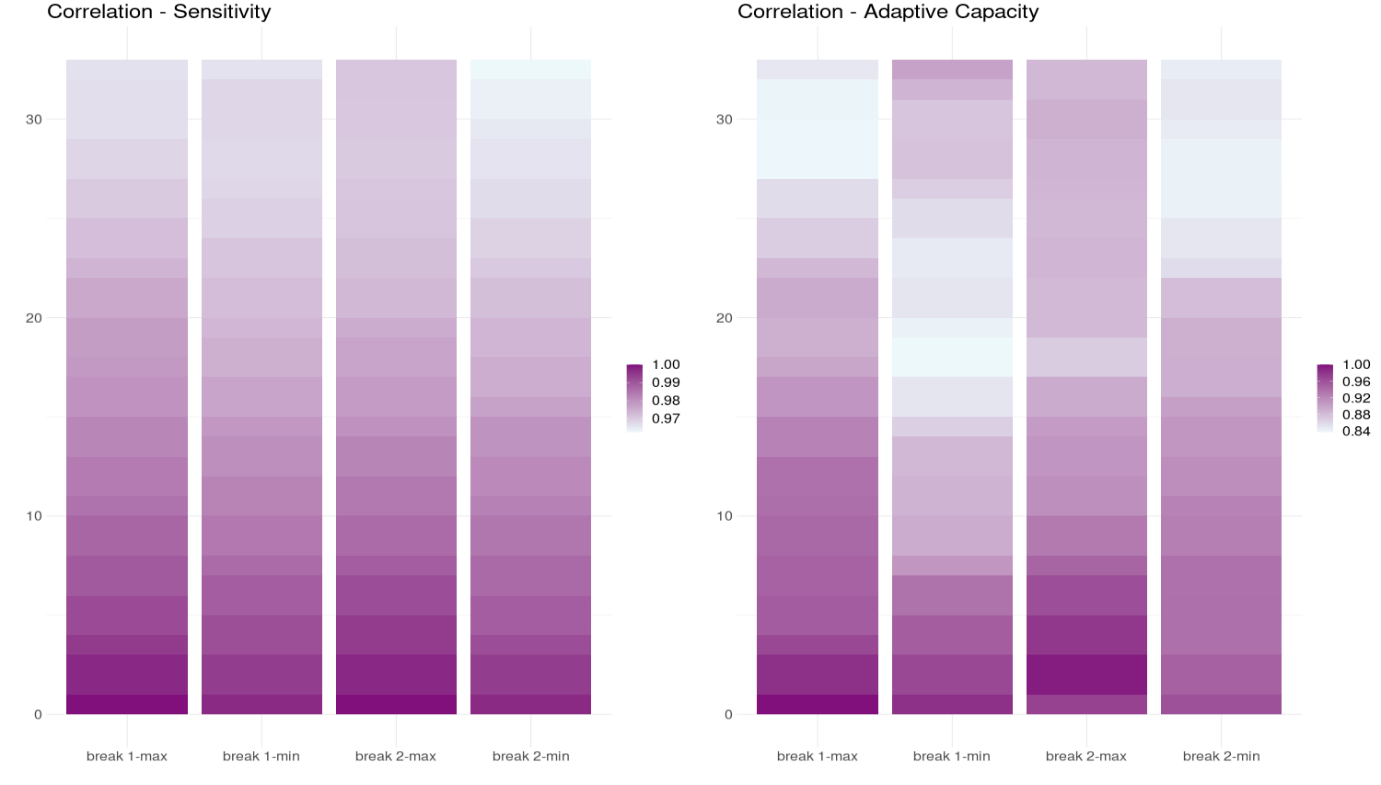


**Figure S5**. Pearson correlation between the sensitivity and adaptive capacity results of the initial scenario and the calculated sensitivity and adaptive scores based on given scenarios. On the y-axis, the amount by which the breaks were moved is shown between 1% and 33%. On the x-as, the multiple scenarios are shown. Namely, moving of the first break towards the maximum (break 1-max), moving of the first break towards the minimum (break 1-min), moving of the second break towards the maximum (break 2-max), and moving of the second break towards the minimum (break 2-max) (Albouy et al., 2020).
